# A Subambient Open Roof Surface under the Mid‐Summer Sun

**DOI:** 10.1002/advs.201500119

**Published:** 2015-05-26

**Authors:** Angus R. Gentle, Geoff B. Smith

**Affiliations:** ^1^ School of Physics and Advanced MaterialsUniversity of Technology SydneyPO Box 123BroadwayNSW2007Australia

**Keywords:** albedo, cool roofs, polymer stacks, sky cooling, urban heat islands

## Abstract

**A novel material open to warm air** stays below ambient temperature under maximum solar intensities of mid‐summer. It is found to be 11 °C cooler than a commercial white cool roof nearby. A combination of specially chosen polymers and a silver thin film yields values near 100% for both solar reflectance, and thermal emittance at infrared wavelengths from 7.9 to 13 μm.

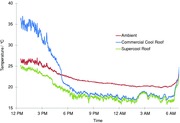

If a surface is under intense sunlight and open to warm air, cooling to below ambient has up to now been an elusive target. The technical goal is super‐cool roofing with thermal performance well above that of existing cool roofs. Stringent spectral requirements at solar and infrared (IR) wavelengths are needed, leading to quite limited choices for suitable coating materials and layer structure. Metal alone, except silver, cannot provide the required level of solar reflectance of above 96% and the thermal emittance of common metals is far too low to cool. Placing silver under a glass or polymer material with very low solar absorptance may cool well as high emittance *E*_r_ results. However, options such as PMMA and most glasses absorb too much solar radiation. Low iron glass and various polyesters including PET absorb very little solar energy if thin, but their IR spectral response is not ideal for this task. Cooling is enhanced if IR spectral response in the thermal emission band involves a mix of moderate reflectance at those wavelengths where the atmosphere irradiates the earth under clear sky conditions and very high absorptance hence emission in the range from 7.9 μm < *λ* < 14 μm, called the “sky window,” which is largely free of incoming radiation as it views the cold of space. Super‐cool surfaces require solar reflectance and sky window absorptance to be close to 100%. The admix of IR reflectance and absorptance in the down‐welling atmospheric radiation band is less stringent but together with the sky window setting determines the value of overall thermal emittance *E*_r_ which dictates the heat output rate at the roof temperature *T*_r_. This radiative rate is an important practical consideration. Its final choice depends on total rate of heat input including the contribution of absorbed down‐welling atmospheric radiation. We concluded that the desired combination of solar and IR criteria could be met using two or more specially chosen polyesters on a silver layer. At the set thickness, their IR absorptance had to be very strong in slightly different sections of the sky window with moderate transmittance at incoming wavelengths. A suitable surface was produced and set up outdoors. It remained subambient throughout a hot summer day including under the peak intense solar intensity of 1060 W m^−2^, with ambient at 27 °C, and high IR intensity from the atmosphere of 400 W m^−2^.

Cool roofing limits total cooling loads in summer^1^, ^2^, ^3^, ^4^, ^5^, ^6^ reduces the severity of the urban heat island (UHI) problem in towns and cities,^7^, ^8^, ^9^ and helps eliminate peak power demand problems from operation of many air conditioners. Added feedback benefits from cool roofs are not yet widely appreciated, but recent reports have shown they are substantial.^10^, ^11^ Examples include ventilation with cooler air and higher performance of adjacent chillers when in cooler air. Adjacent cooler air also lowers temperature gains from convective exchange for a subambient roof. Air above established roofs is much warmer in the daytime.

The thwarting by solar absorption of daytime radiative cooling to a subambient surface seemed until 2014 an insurmountable barrier. Thus, it received little scientific interest over the last 40 years. Trombe in the 1960s was a pioneer, but his surface was not in direct sun.^12^ This and the 1990s daytime study by Nilsson and Niklasson^13^ used one or more outer polymer foils to block convective gain. Reflective pigments such as TiO_2_, ZnS, and ZnO doped into IR transmitting polymer such as poly­ethylene did not achieve net cooling under the sun.^13^ The first report of subambient daytime cooling appeared late in 2014.^14^ A thin polyethylene cover to suppress convective exchange but transmit IR was used separated from a stack of two thin oxides deposited over 200 nm of silver on silicon. The demonstration here is the first using polymers and an open surface, and is suited to basic roofing. Spectrally suitable polymers happened to be available commercially as coextruded combinations of many bilayers.^15^, ^16^ This has an additional advantage as it acts as an all‐dielectric mirror and reflects better than metals at blue wavelengths. The best stacks let through some NIR solar energy and a considerable amount of atmospheric radiation so modification was needed.

Such mirrors use multiples of birefringent polymer pairs, one with high index and one with low index, for example polyethylene terephthalate (PET)/naphthalene dicarboxylate and polyethylene napthalate (PEN)/THV (a 3 m fluoro‐thermoplastic containing tetrafluoroethylene, hexafluoropropylene, and vinylidene fluoride). Further spectral details and the layer structure of polymer mirror stacks, in which pair thicknesses are graded to set the breadth of spectral and angular response, are in ref. 15. The product used Vikuiti Enhanced Specular Reflector (ESR) is all polyester and believed to consist of PET/ECDL pairs with ECDEL a Kodak copolyester using 1,4‐cyclohexane dicarboxylic acid, 1,4‐cyclohexane dimethanol, and polytetramethylene ether glycol. The overall thickness of this polymer mirror is 67 ± 4 μm and consists of 300 layers of each polymer plus PET outer layer,^17^ so average thickness per layer is near 110 nm. The average final density of the stack is 1.29 gm cc^−1^.^18^ Assuming the bulk density ratios of PET (1.38 gm cc^−1^) to ECDEL (1.13 gm cc^−1^) persists in the final stack, the layer thickness for PET averages 141 nm and ECDEL 79 nm. These thicknesses are, however, graded with individual layer thicknesses on one side of the stack about 1/4 that of those on the opposite side.^15^ This feature extends the high reflectance band to 1 μm. PET is uniaxial with vertical component of refractive index 1.51 and in‐plane index 1.66,^17^ while ECDEL is isotropic with index 1.52. Layer thickness profile and in‐plane index differences provide most of the very high solar reflectance from 0.4 to 1 μm. These two polymers in combination plus each one's total thickness of 42.3 and 23.7 μm for PET and ECDEL, respectively, also yield the required strong sky window absorptance, and moderate transmittance at down‐welling IR bands. The bare stack thus needed to have its solar reflectance raised in the NIR, while its transmitted IR had to be reflected. Simply adding a bottom silver coating achieved both solar and IR goals.

The complete solar‐IR spectral reflectance of the resulting surface is in **Figure** **1**. The *R*(*λ*) value is very low as desired across the sky window zone. The silver reduces heat gained from the atmosphere and raises the Air Mass 1.5 albedo to 0.97. The solar reflectance was also measured from normal incidence out to 85° incidence. Another remarkable feature of this coated polymer stack is that this part of the spectrum in Figure 1 is almost constant over this wide angle range. These data are plotted in the Supporting Information.

**Figure 1:**
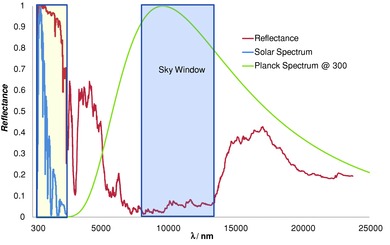
Spectral reflectance of the super‐cool roof material across the solar and thermal infrared showing key cutoff wavelengths including the boundaries of the sky window (blue shading) and the solar spectrum (yellow shading). Spectral response in the unshaded zones is where down‐welling sky radiation occurs. The solar spectrum (blue) and Planck spectrum at 300 K (green) are plotted for reference.

The common feature between this and the recent^14^ subambient study is a very high solar reflectance (*R*_sol_), so for 1000 W m^−2^ incident around 30 W m^−2^ of solar energy is absorbed. However, output of thermal radiation at roof temperature *T*_r_ in this study must remove not only the absorbed solar energy, but heat from three other sources; direct convective gain from the air once *T*_r_ drops below ambient temperature *T*_a_, heat produced by absorption of incoming atmospheric radiation, and the parasitic heat input from supporting structures. Thus, spectral response in the IR for both clear and average sky conditions needs consideration.

This surface meets the main prerequisites for subambient cooling in the daytime with its *R*_sol_ and thermal emittance from 7.9 to 14 μm both close to 100%. Finally, one must consider if this surface has a suitable response to down‐welling thermal radiation which is largely confined to wavelengths where the atmosphere has high emittance arising from water vapor and carbon dioxide. The spectral emittance across the sky window averages 0.96. The combination of this value with its albedo of 0.97 will be hard to improve upon in practice. Very high values of one or the other are well known but not the combination. For example, an admix of two common light‐weight nanoparticles, SiO_2_ and SiC, in polyethylene on aluminum gave a sky window emittance just below 100%,^19^ but *R*_sol_ was well below 0.97. The oxide multilayer system of^14^ also had albedo of 0.97, but its sky window emittance was well below 0.97 being near 0.6. This was adequate under a cover to cool to subambient in the daytime in conditions presumed to be for a Californian winter, as it was combined with good reflectance of the down‐welling IR radiation.

The optimum spectral approach to reflection of down‐welling thermal radiation is an important issue for super‐cool roofs but not for current average roof albedos, nor existing cool roofs whose albedo typically lies between 0.70 and 0.85. As a general rule, roofs that cannot achieve daytime subambience should have black body emittances *E*_r_ above 0.8 so as to maximize emitted radiation. Then, spectral variations across the thermal IR cannot be large. For these existing roof products the common approximation for absorption of down‐welling radiation intensity *P*_A,DW_ given below in Equation (1) is adequate. The impact of the sky window is to reduce the incoming intensity below that of a black hemisphere at *T* = *T*_a_ to that at a lower effective temperature *T*_sky_:

(1) PA, DW =ErσT sky 4

The thermal radiation power emitted given by Equation (2) is higher than *P*_A,DW_ as long as roof temperature *T*_r_ is above *T*_sky_, allowing subambient net cooling in the range *T*_a_ > *T*_r_ > *T*_sky_ to arise on clear nights if parasitic and convective heat gains are small enough.

(2) $$P_{r,\mathrm{out}}=E_{r}\sigma T_{r}^{4}$$

*E*_r_ and *P*_A,DW_ are formally derived in the Supporting Information from the spectral and directional IR response of a surface. The IR spectral response measured at six incident angles ranging from 15° to 85° is plotted in the Supporting Information which also explains how these data were used to estimate the hemispherical emittance and provide an accurate estimate of the reflectance of down‐welling radiation. The *E*_r_ value was calculated to be 0.63. This accurate treatment shows that Equation (2) still applies, but the approximation to *P*_A,DW_ of Equation (1) breaks down if the radiating surface has large swings in spectral response across the Planck range. Spectral density of down‐welling radiance involves directional and spectral variations in incoming intensity. It depends on humidity and cloud distribution. For clear skies only variation with angle to the zenith^5^ is needed. An essential consideration in our surface thermal response models is that the atmospheric spectral density plot itself is sensitive to direction. It is quite different when viewing the sky near the horizon compared to near the zenith. This spectral change plus the higher intensity near the horizon impacts optimum surface design.

It is common practice to lump absorbed and outgoing IR flows together into one “cooling rate,” but here Equation (2) is the actual “cooling rate.” It must account for all heat gain mechanisms, not just Equation (1). The “lumping” approach has led to a focus on ideal sky window selectivity in which nearly all down‐welling radiation is reflected and absorptance is maximized across the sky window. This limit means *E*_r_ ≈ 0.3 and a very low cooling rate. A significantly higher *E*_r_ is actually better in most practical situations including when *T*_r_ values are just below ambient, as when open to air as in this study, and for other than clear sky conditions. This is because it allows a faster rate of cooling for realistic total heat inputs. The absence of a convective barrier limits our [*T*_a_ – *T*_r_] value to of order 3 °C for peak midday conditions on a clear summer day, and to 7 °C at night.

The cost of most emittance being confined to the sky window means it may take a very long time to get to high enough [*T*_a_ – *T*_r_] values to justify such a spectral response. Its low pumping rates mean the mass to be cooled has to be small, and convective gain must be suppressed.^20^ Our multiple day results below indicate that an intermediate reflectance of down‐welling radiation works well for average skies. For all cool roofs in current use, solar reflectance is not high enough to consider a lowering of *E*_r_. This is why the most commonly used figure of merit for cool paints, the SRI index,^21^ is best when *E*_r_ is as high as possible. However, the basic SRI model is not a good quality guide once daytime subambient capability exists because of the associated IR spectral variations as in Figure 1.

We now present outdoors thermal data for a clean, unprotected new surface, and one aged over several days in a polluted outdoor summer environment for the assessment of the impact of the buildup of dust and grime. Reduction of albedo is a common concern for cool roofs and self‐cleaning, or enhanced water and rain cleaning is thus of growing interest.^22^, ^23^ Extensive dew formation is inevitable for a super‐cool roof and dew‐drops precipitate dirt. This roof site being 25 m above a busy city transit road was a stern test. Results show that excellent thermal performance can be maintained.

**Figure** **2** compares outdoor temperatures of the corrugated metal, white cool roof with that of the super‐cool polymer multilayer on silver. The painted roof had been outdoors for 3 years. *R*_sol_ had dropped to 0.74, from 0.77 when new, and emittance was 0.90. Data include a period with peak solar insolation of 1060 W m^−2^. The solar and atmospheric down‐welling intensities on the samples over the course of this experiment are plotted in Figure S1 of the Supporting Information. The experimental arrangements are described below. *T*_a_ peaked near 27 °C that day. The one other subambient daytime study^14^ was in much cooler 18 °C air and under peak fluxes between 850 and 900 W m^−2^. This test had up to 25% higher solar intensity, no convection suppressing barrier, and summer‐time down‐welling IR intensities near 400 W m^−2^. Achieving (*T*_a_ − *T*_r_) = 2 °C under 1060 W m^−2^ with no convection shield is a new benchmark and 11 °C below a quality commercial cool roof is a significant leap in thermal performance.

**Figure 2:**
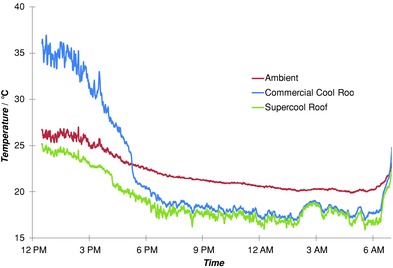
A comparison of surface temperatures on a clear summer day of two different open “cool” surfaces with that of ambient air. The super‐cool system remains subambient throughout the day, while the commercial cool roof is 9 °C above ambient and 11 °C above the super‐cool roof under peak solar conditions and 27 °C ambient air.

Extended outdoors exposure of this surface did not stop it remaining below summer ambient most of the time. Nine consecutive mid‐summer days of data for the super‐cool surface and the commercial cool surface are in **Figure** **3**, which shows the differential to ambient after some soiling. A plot of absolute temperatures is in the Supporting Information. Over a period of about 2 h near midday, a small rise of 0.5–1.5 °C above ambient occurred. It then fell below ambient for the rest of the day reaching 7 °C below ambient at night. *T*_r_ always remained below the normal cool roof by around 11 °C when surface temperatures peaked. This difference falls to 1–2 °C at night.

**Figure 3:**
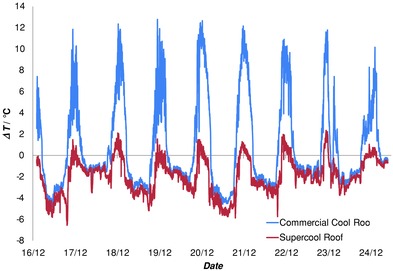
Performance of the super‐cool material and the side‐by‐side existing commercial cool roof during extended exposure near a main transit city road over a 10 d summer period. Temperature of each is plotted relative to ambient.

As the daytime *T*_r_ value remains at or below ambient the absolute change in the super‐cool roof from the midday maximum to night minimum is just 7 °C larger than the diurnal change in ambient. In contrast, this excess change for the existing cool roof is a much larger 18 °C. An average roof with medium albedo will undergo an even bigger excess change of up to 60 °C. The larger changes are not gradual and most coincide with the rapid changes in solar intensity that occur in the morning and evening. Minimizing such thermal swings of building elements and coatings is a valuable bonus. It will damp thermal stresses and crack evolution.

This material is suited to large‐scale production and application to some roofing materials. Refinements needed include a means of making the reflectance diffuse to avoid glare without raising solar absorptance. This may be possible by adding a high transmittance but diffusing polymer layer^24^ or via surface microstructure. Materials approaches to transforming normal roofs to super‐cool roofs have been outlined with a practical demonstration. They combine three spectral elements; albedo and sky window absorptance both close to 100% and a compromise on reflectance and absorptance of atmospheric radiation for a sufficient rate of heat removal. This last aspect will depend on local climate and building design.

## Experimental Section

Knowing PET plus another polyester with a slightly longer IR peak absorption band on silver could provide suitable solar and IR responses our search for the best polyester combination led to the possibility of using a two‐polyester solar mirror from 3M.^16^, ^18^ Its solar reflectance and transmittance was first measured using a Perkin Elmer (PE) Lambda950 spectrophotometer, and its IR spectral reflectance and transmittance to 25 μm with a Hitachi 270‐30 Infrared Spectrometer. The angle of incidence dependence of IR reflectance was measured by placing a PE variable angle IR reflectance attachment in the Hitachi Spectrometer. The angle dependence of reflectance at solar wavelengths was measured with a Woollam spectroscopic ellipsometer. To test that adding a silver coating would yield the desired *R*_sol_ above 95% and also the desired IR spectral selectivity, the combined response was modeled. An area of 190 × 190 mm of polymer mirror was then sputter coated with 200 nm of silver. This sample gave the spectrum in Figure 1. It was set up outdoors, alongside an existing small structure specifically designed for studying cool roofs as previously reported.^1^, ^25^ The sample was mounted horizontally on a polystyrene block, flush with the surface, under which a small cavity was cut out to allow room for mounting the temperature sensors. In this location (Sydney, Australia) at the time of year peak sun was at 13° to the vertical, which defines the angle of peak beam solar radiation incident in this experiment.

The cool roof was factory coated on a corrugated thin metal panel and supplied by Bluescope steel. Both were free of shadowing, with a clear view of the sky. A Middleton pyranometer provided accurate global insolation data, while a periodically switched shadow band over a silicon solar sensor provided the diffuse solar fraction. An accurate pyrgeometer MS‐202 from Eko instruments recorded down‐welling IR intensities. Small‐temperature sensors (DS18b20 digital thermometers) were attached to the underside of both surfaces to avoid direct solar heating with copper tape to ensure good thermal contact. Other weather data needed were recorded onsite with a WS‐3081 weather station. It included air temperature, humidity, dew point, plus wind speed, and direction. Solar, IR, and weather sensors were mounted behind the two surfaces of interest on the south with the sun in the north in Australia. The solar and atmospheric intensities recorded during the acquisition of Figure 2 were plotted in the Supporting Information which also contained a plot of the absolute temperatures of the two roof materials and ambient over the 9 d experiment.

## Supporting information

As a service to our authors and readers, this journal provides supporting information supplied by the authors. Such materials are peer reviewed and may be re‐organized for online delivery, but are not copy‐edited or typeset. Technical support issues arising from supporting information (other than missing files) should be addressed to the authors.

Supplementary
