# Supplementary material for: A Subambient Open Roof Surface under the Mid‐Summer Sun
Source: Adv Sci (Weinh). 2015 May 26;2(9):1500119. doi: 10.1002/advs.201500119 (PMC5115392; doi:10.1002/advs.201500119)
Supplement: Supplementary file 1 — Supplementary [file ADVS-2-0c-s001.pdf]

Copyright WILEY-VCH Verlag GmbH & Co. KGaA, 69469 Weinheim, Germany, 2015.

ADVANCED SCIENCE
Open Access

Supporting Information

for *Adv. Sci.*, DOI: 10.1002/advs.201500119

A Subambient Open Roof Surface under the Mid-Summer Sun

*Angus R. Gentle and Geoff B. Smith**

Supporting material

Performance sensitivity to small changes in solar reflectance

Small shifts in solar reflectance in the range from 0.90 to 1.0 have a significant relative impact on the performance of super-cool roofs and related materials which can out-perform existing cool roofs. Thus it is important to have a well-defined standard approach and accurate surface spectral reflectance data for opaque samples when evaluating different material possibilities. Standardized comparison of how different materials respond to solar energy relies on the relevant materials spectrum like Figure 1, weighted by a normalized standard solar spectrum. The most common choice involves the Air Mass 1.5 (AM1.5) solar spectrum but even for this slightly different approaches have been used which can lead to significant changes in albedos near 1.0. The spectrum of Figure 1 weighted by the NFRC_300_2003 : ASTME892 standard solar spectrum gave albedo of 0.963, while the more recent ISO9050 : ASTME891 standard AM1.5 spectrum gave 0.978. This difference arises because these standards differ slightly in the UV-blue zones while these polyesters have a sharp cut-off near 400nm. The difference in R_{sol} between standards would mean a difference of 15 Wm^{-2} in the amount of solar heat that had to be removed radiatively under a peak summer sun which is a large relative shift given the total solar heat gain is so low and net radiation outflows available to remove solar, convective and any parasitic heat input are under 80 Wm^{-2} for this open roof.

Additional environmental energy and roof temperature data

The most important environmental data impacting on the observations presented are ambient temperature, horizontal total solar intensity, and intensity of down-welling atmospheric radiation. Ambient temperature over the

day was in **Figure 2**. The associated solar and atmospheric irradiance intensities over the same day are plotted in (**Figure S1**).

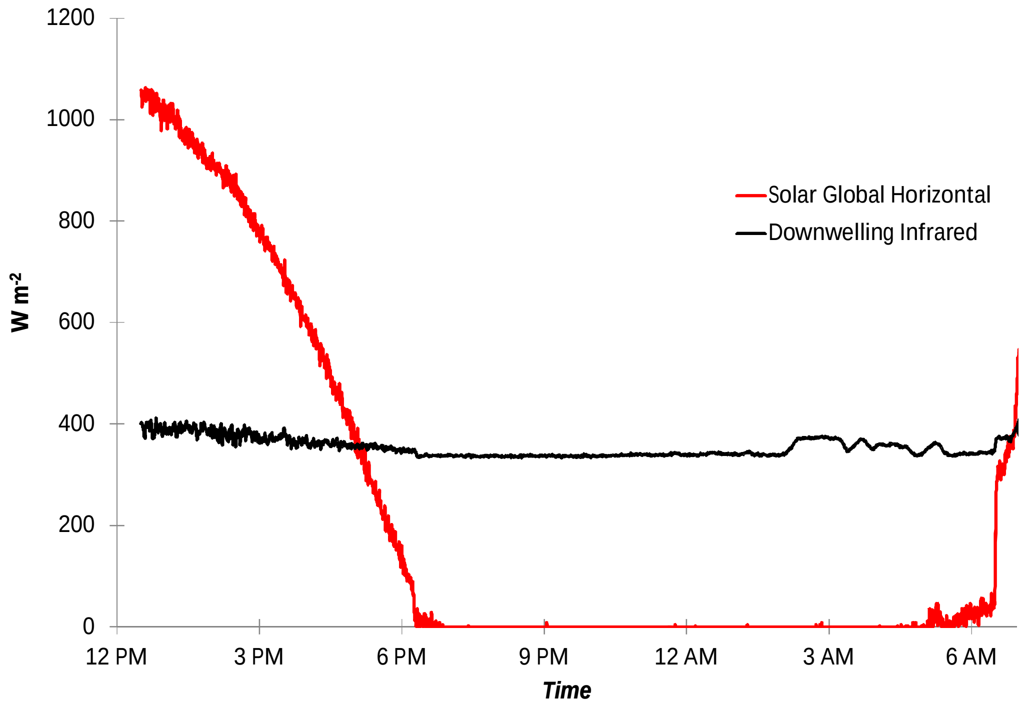

Figure S1. Measured incoming environmental radiant intensities during the experiment with a fresh sample. Solar and down-welling infra-red are both shown.

Dew point and humidity variations influence down-welling intensity. The way this affects atmospheric spectral emittance is modeled later. Wind speed impacts on convective exchange, and was recorded. For normal and standard cool roofs in the daytime it adds to cooling but if daytime sub-ambient cooling occurs, and more generally at night for roofs in general, it adds to heat gain. Detailed wind speed data is available as recorded during all experiments reported and was low at < 4 ms^{-1} all day when data in Figure S1 and Figure 1 were recorded, and during most of the 9-day data acquisition. The surface and ambient

temperatures recorded in the 9-day experiment are plotted in **Figure S2.** The differential temperature to ambient of Figure 3 were derived from this data. It is clear from Figure S2 that the supercool roof is nearly always very near or below ambient while the normal “cool roof “ is well above ambient during the day. It is also interesting to compare the two at night. Due to the similar emittance within the sky window of the open cool roof and super-cool roof they achieve similar night temperatures, though the super-cool, IR selective roof does get a little cooler. This occurs despite differences in other sections of the IR. A convection suppressing cover would alter this night difference by reducing the heat gain from the much warmer night air and while both get even cooler the difference apparent in Figure S2 between both materials will in general widen further. The section following discusses this from a modeling perspective.

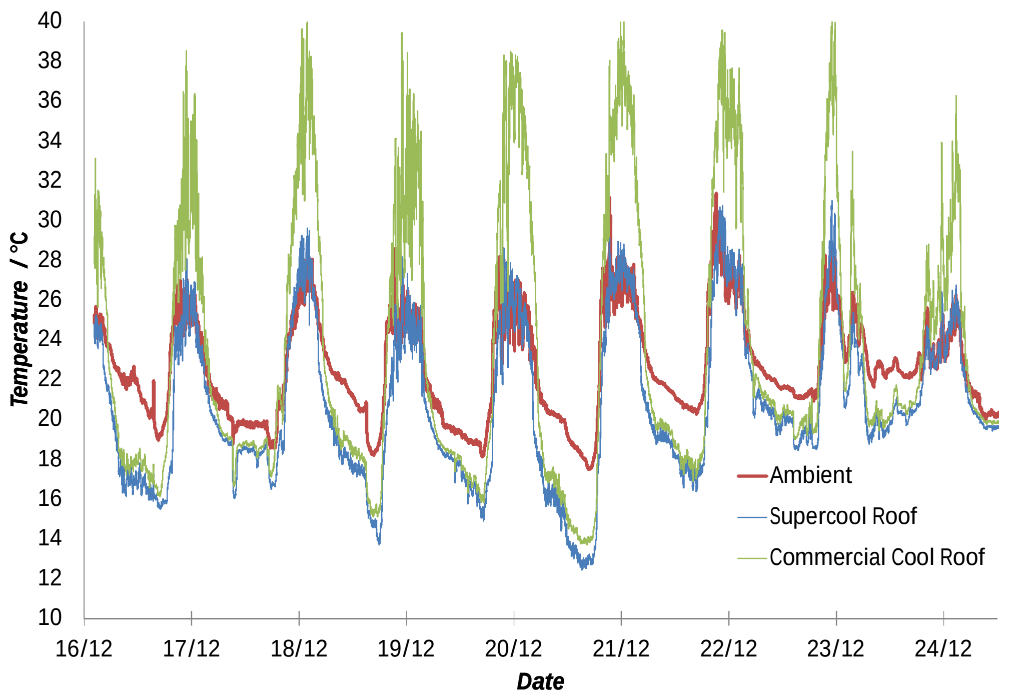

Figure S2. Measured ambient and roof temperatures over the 9 day period in mid-summer.

Spectral and angle of incidence data

Since solar position in the sky and intensity varies throughout a day and seasonally, and at any instant the clear sky incoming atmospheric radiation spectrum and intensity changes with angle to the zenith, accurate thermal modeling requires solar and IR spectral reflectance of the surface to be known at each angle of incidence. Solar spectral and angular data acquired and used for such modeling on each surface used in this study is in **Figure S3** and IR data in **Figure S4**. The near constancy of the solar reflectance spectrum with incidence angle at a very high albedo value is seen in Figure S3. This simplifies the sun’s thermal influence in simulation studies for this surface, and most importantly maintains a high albedo for most sun locations, including all sun positions where solar intensity can heat a surface. The atmosphere’s influence is more complex as incoming IR rays come from all sky directions. Each one’s intensity can be calculated for any humidity level as explained in the next section. The model for surface absorption response following involves both this complex IR intensity profile and angular material data such as that in Figure S4.

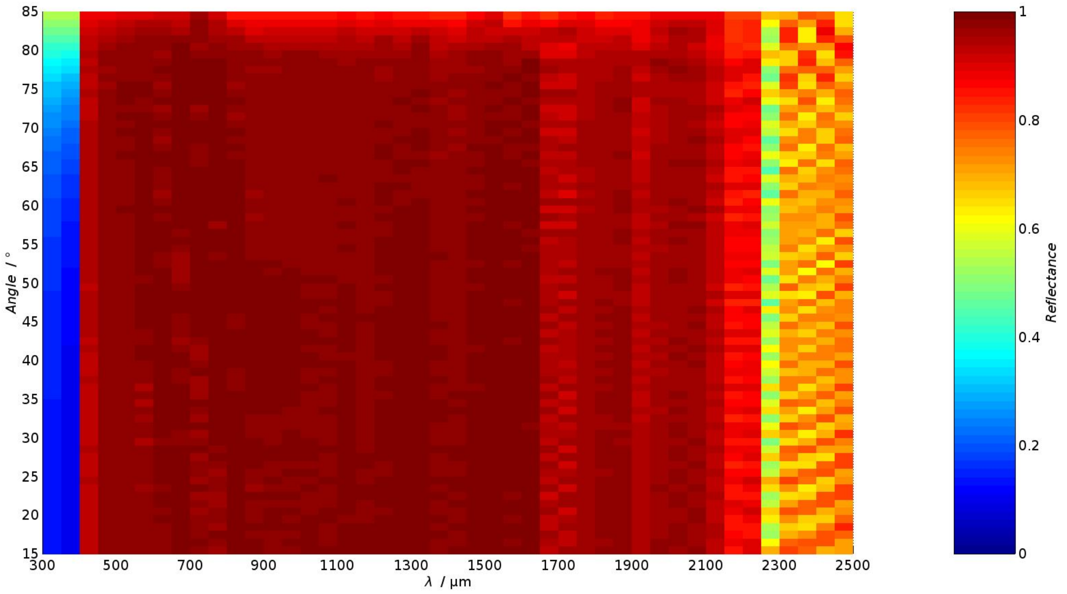

Figure S3. Angular variation of reflectance across solar wavelengths. Specular reflectance was measured from 15-85° at 1° steps using a V-VASE Woollam Ellipsometer in Reflectance Mode.

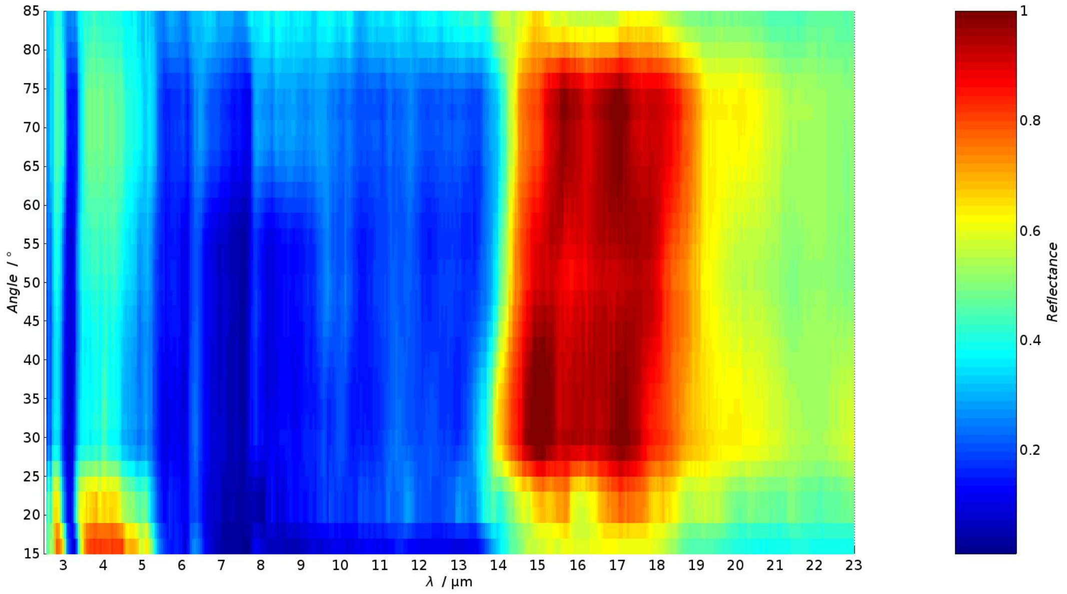

Figure S4. Angular reflectance at infra-red wavelengths for incident angles ranging from 15° to 85°(15,20,30,50,70,85°). The absorptance weighted by the Planck spectrum at 300°K for each of these angles yields the emittance values listed in **table 1**

Table 1. Angular dependence of emittance @ 300K

Angle	15	20	30	50	70	85
Emittance	0.74	0.67	0.60	0.62	0.58	0.58

The data in Figure S4 can be used in the equation for E_r in the next section to estimate hemispherical emittance. This calculation gave $E_r = 0.63$.

Complete models of thermal emission and heat gain from down-welling radiation

The calculation of radiation emitted from the surface, hence heat-pumping rate is given by **equation (s1).** The accurate calculation of the absorbed down-welling atmospheric radiation (**equation s2**) under all sky conditions is needed if spectral selectivity exists in the IR as in Figure 1. Equation (s2) does this. It weights at each incoming direction the normalized atmospheric radiation spectrum given by atmospheric spectral emittance $E_{atm}(\theta,\lambda)$ with the Planck black body emission spectrum $P(\lambda,T_a)$ at atmospheric temperature T_a, and the spectral reflectance of the surface at that angle. This explicitly allows for the substantial variation in incoming spectral density as incoming ray direction to the zenith shifts down from the vertical. This angle to the zenith is given by θ. A uniform hemisphere has been assumed for equation (s2) by dropping azimuth (ϕ) dependence in $E_{atm}(\theta, \phi, \lambda)$ as for clear skies.

$$P_{r,out} = \int_0^{\pi/2} d(\sin^2\theta) \int_0^{\infty} d\lambda P(\lambda, T_r) E_r(\theta, \lambda) = E_r \sigma T_r^4 \quad \text{(s1)}$$

$$P_{A,DW} = \int_0^{\pi/2} d(\sin^2\theta) \int_0^{\infty} d\lambda P(\lambda, T_a) E_{atm}(\theta, PWV, \lambda) [E_r(\theta, \lambda)] \quad \text{(s2)}$$

The strong humidity dependence of down-welling intensity, and of its spectral distribution, has also been introduced into equation (s2) by replacing $E_{atm}(\theta, \lambda)$ by $E_{atm}(\theta, PWV, \lambda)$ using precipitable water vapour height PWV which is easily linked to dew point T_{DW} or relative humidity RH if desired. PWV has a strong influence on the variation of spectral density with (θ, ϕ). The way this is linked to direction for uniform skies is from **equations (s3) and (s4)** together, with $\Gamma_{atm,z}(\lambda) = \Gamma_a(0°, PWV, \lambda)$ the atmospheric transmittance at the zenith for each wavelength. The θ dependence arises from the atmosphere's attenuation depending exponentially on its thickness. This yields the variation with θ in equation (s4).

$$E_{atm,z}(PWV,\lambda) = 1 - \Gamma_{atm,z}(PWV,\lambda) \quad \text{(s3)}$$

$$E_{atm}(\theta, PWV,\lambda) = 1 - \Gamma_{atm,z}(PWV,\lambda)^{1/\cos\theta} \quad \text{(s4)}$$

PWV ranges from 8 to 11 mm in humid and seaside locations and is below 5 mm in desert locations. The associated incoming spectral intensities are quite different, which leads to major differences in the optimum IR surface spectra for best cooling. Thus typical local humidity levels should be considered when assessing the benefits of a particular surface's spectral response or in complete analysis of experimental data. We have previously published an example of this from modelling and from experimental comparison at night of the relative cooling rates of a surface with a high emittance of 0.92 relative to one which is sky window selective[s1]. The latter consisted of a polyethylene sheet doped with nano-phononic resonant nanoparticles on aluminum[s2] . Only at high humidity levels did the sky window selectivity sample

yield a higher cooling rate, and only then once surface temperature had fallen to more than 6°C below ambient. For desert locations this cross-over requires even greater cooling to around 10°C below ambient.

At first it seems counter-intuitive for high reflectance of incoming sky radiation plus high emittance in the sky window to work best when humidity is higher, as then the sky window is also less transmitting and more radiation is incident. The reason is however simple as the sky window selective surface performance has weaker sensitivity to changes in incoming IR radiation as it absorbs very little. In contrast a high emittance surface is very sensitive to a large drop in irradiance as it absorbs most of whatever is incident. As a high emittance gives a much superior radiation rate at any surface temperature to that from a strong sky window emitter the latter can only cool faster by absorbing much less of the incoming energy. Drier atmospheric conditions make this unlikely until the surface is so cold relative to ambient (as a guide [$T_a – T_r$] > 10°C) that superior radiant output from the high emittance surface is lower than the difference in absorbed IR between each. Though the surface in this paper is sky window selective it lies optically between these down-welling reflectance extremes.

For daytime cooling it must be noted that this choice to reflect or absorb down-welling radiation only becomes relevant if super-cooling to near or below daytime ambient is possible. This means in the daytime a pre-requisite to having a much reduced emittance is a very high albedo and a very high sky window emittance. Broad-band high emittance is thus preferred unless cooling below ambient is likely to be achieved. Very high sky window emittance occurs for both good sky window

spectral selectivity and for broad-band high emittance surfaces. The spectral design for handling of incoming IR radiation can be considered more generally if night cooling only is the goal as then (T_a – T_r) can get to 7° or more, and get even cooler if convection suppressant IR transmitting covers are used, but the amount of heat needed to be pumped away to get there must be included in the calculations. In the experiments reported here an intermediate approach was taken to reflecting some of the incoming sky radiation, and it worked well in the daytime and at night without convection suppression. Super-cooling of roofs in daytime is now a proven possibility. It could in due course add much to energy savings in buildings and to counter-measures to the UHI effect, so optimizing spectral response for a given climate zone is an issue which deserves further attention.

Surface spectral response and steady state roof temperature T_r

The steady state roof temperature occurs when heat output and input are in balance. If sub-ambience is achieved the only output mechanism is thermal radiation as given by $P_{r,out}$. Heat input has then four components; (a) solar heating $P_{sol}= A_{sol,r}\Phi_{solar,r}$ (b) absorbed down welling $P_{A,DW}$ (c) parasitic heat gain P_{ps} from the supporting building (d) convective exchange with local air given by $U(v_W)$ $[T_a – T_r]$ which varies with wind speed v_W. $A_{sol,r}$ is the roof surface solar absorptance and $\Phi_{solar,r}$ the solar insolation intensity incident on the roof. Both depend on roof tilt and orientation. All vary in time but the largest dynamic influence is the change in solar flux. Parasitic gains may also involve a time constant from heat storage in the thermal mass of the roof and to some extent the supporting building. If the latter influence is slow enough or weak enough the steady surface temperature can be found from **equation (s3).**

$$P_{r,out} = A_{sol,r}\, \Phi_{solar,r} + P_{A,DW} + P_{ps} + U(v_W)\,[T_a - T_r] \qquad (s4)$$

[s1] A. Gentle, G. Smith, Performance comparisons of sky window spectral selective and high emittance radiant cooling systems under varying atmospheric conditions, *Proc. AUSES Solar2010 Conference*, http://www.auses.org.au/information-portal
[s2] A.R. Gentle, G.B. Smith, Radiative heat pumping from the earth using surface phonon resonant nanoparticles, *NanoLetters*, **10**, 373-379 (2010).
